# Transesophageal Electrophysiological Study in Children Under 12 Years of Age with Asymptomatic Wolff–Parkinson–White Syndrome

**DOI:** 10.3390/biomedicines14020279

**Published:** 2026-01-27

**Authors:** Gabriel Cismaru, Marius Muresan, Alina Negru

**Affiliations:** 1 Fourth Department of Internal Medicine, Cardiology-Rehabilitation, “Iuliu Hatieganu” University of Medicine and Pharmacy, 400012 Cluj-Napoca, Romania; 2 Department of Anaesthesia, Monza Hospital, 400347 Cluj-Napoca, Romania; marius.muresan@reginamaria.ro; 3 Department of Cardiology, University of Medicine and Pharmacy “Victor Babes”, 300041 Timisoara, Romania; eivanica@yahoo.com; 4 Rocordis Heart Center, Cardiology and Cardiovascular Surgery Hospital, 300041 Timisoara, Romania

**Keywords:** accessory pathway, WPW syndrome, transesophageal, electrophysiological study, sudden death, high risk

## Abstract

**Background/Objectives**: Patients with WPW syndrome have a risk of sudden cardiac death that can be assessed using an electrophysiological study. In symptomatic patients, the preferred route is intracardiac, whereas in asymptomatic children, transesophageal. Our study aimed to evaluate the risk using a transesophageal study, considering a threshold age of 12 years for sedation. **Methods**: We investigated 41 asymptomatic WPW children with a mean age of 12.5 ± 4.4 years (range 1 to 18 years old), with 48.8% being male. We determined three values: (1) the accessory pathway effective refractory period (APERP), (2) the minimal cycle length demonstrating 1:1 conduction through the accessory pathway, and (3) the shortest RR interval between two consecutive pre-excited beats during atrial fibrillation. **Results**: Children under 12 years had a mean age of 7.5 ± 2.5 years, while those over 12 years had a mean age of 15.5 ± 1.9 years. Sedation was administered exclusively to children under 12 years of age. Orthodromic reentrant tachycardia was induced in four children, and atrial fibrillation was induced in 14 children. Comparing the group under 12 with the group over 12, the mean APERP was 296 ± 38 ms vs. 286 ± 45 ms (*p* = 0.48), the average 1:1 conduction over the accessory pathway was 287.3 ± 41 ms vs. 282 ± 46 ms (*p* = 0.71), and the average shortest pre-excited RR interval during atrial fibrillation was 280 ms vs. 262 ms years (*p* = 0.75). **Conclusions**: Asymptomatic children under 12 years of age showed a lower incidence of inducible atrial fibrillation. They had accessory pathways with reduced risk, except one, and no children under 12 years underwent catheter ablation.

## 1. Introduction

Adults and children with Wolff–Parkinson–White (WPW) syndrome have a risk of sudden cardiac death due to atrial fibrillation with a fast conduction through the accessory pathway leading to ventricular fibrillation [1]. Sudden cardiac death may be the initial clinical manifestation of WPW syndrome in asymptomatic children. Reports indicate that 12% of patients with WPW who experienced cardiac arrest were previously asymptomatic, with no palpitations, dyspneea or syncope [2,3].

Compared to adults, children have distinct electrophysiological characteristics of both the accessory pathway and the atrioventricular node, along with a significantly higher prevalence of multiple pathways, which may influence prognosis and recurrences after catheter ablation [4]. In children aged 5 to 18 years, the prognosis of asymptomatic ventricular pre-excitation, when discovered by chance, is not as favorable as previously believed as nearly fifty percent of children experiencing cardiac arrest due to Wolff–Parkinson–White syndrome had no significant preceding clinical manifestations [5]. Multiple pathways and short effective refractory periods are recognized as independent predictors of potentially life-threatening arrhythmic events [6].

Risk classification for asymptomatic WPW children has been recommended to identify those at elevated risk of sudden death and thus eligible for ablation. The criteria for ablation include inducible orthodromic tachycardia and fast antegrade conduction via the accessory pathway [7]. The transesophageal electrophysiological study offers a fairly noninvasive method for risk stratification in asymptomatic children, while the intracardiac electrophysiology study involves additional risks, including bleeding, vascular damage, pericardial effusion, AV block, and a prolonged recovery period, being reserved for symptomatic children [8]. Therefore, we set out to determine risk in asymptomatic WPW children using transesophageal electrophysiological study with and without sedation using a threshold age of 12 years.

## 2. Materials and Methods

### 2.1. Patients

We included in this retrospective study 41 consecutive children with pre-excitation on their electrocardiogram, without a history of tachycardia or other cardiac symptoms, who underwent transesophageal electrophysiologic testing from April 2022 to December 2025 at the Monza Ares Hospital and Napoca Clinic, Cluj-Napoca, Romania. All children had a Holter ECG and/or a stress test before the study, showing persistence of conduction through the accessory pathway up to the maximum recorder rate or absence of abrupt disappearance of the WPW pattern during exercise. Prior to the transesophageal study, informed consent was obtained from the parents.

### 2.2. Electrophysiological Testing

Transesophageal studies were performed under conscious sedation for children under 12 years of age and without sedation for those above 12 years. After local anesthesia with 2% lidocaine, the pacing lead was introduced through the nasal or oral route into the middle esophagus. Pacing was started after we obtained sensing of the electrical signal from the left atrium. We used a Micropace III stimulator (Sidney, Australia), connected to a FiAB (Vicchio, Italy) 5 Fr transesophageal quadripolar or bipolar probe, and we delivered impulses of 10 to 25 mA with a duration of 10 ms. Capture was confirmed on a 12-lead ECG. The esophageal electrogram was recorded on the Micropace III screen, and surface ECG lead signals were recorded on paper at a speed of 25 or 50 mm/s. The patients underwent a protocol of single extrastimuli pacing as well as rapid atrial pacing in the baseline state. The following values were measured: antegrade effective refractory period of the accessory pathway (APERP), antegrade conduction through the accessory pathway with a minimum 1:1 atrial cycle length, and the shortest pre-excited RR interval during atrial fibrillation. The duration of the procedure was five to 10 min. When sedation was needed, it took 10 to 20 more minutes to place the peripheral venous line and inject sedatives. All patients went home after the electrophysiological study; none were hospitalized. Catheter ablation followed the transesophageal EP study if 1 or more of the following values were obtained in the basal state: inducible orthodromic reentrant tachycardia, APERP < 250 ms, antegrade AP min 1:1 < 250 ms, or the shortest pre-excited RR interval during AF < 250 ms.

The effective refractory period of the accessory pathway was determined by pacing the left atrium at a cycle length of 500 ms, with extrastimuli starting from 400 ms with 10 ms decrement for each beat until the effective refractory period (ERP) was obtained. Accessory pathway ERP was defined as the longest A1–A2 interval that failed to conduct in the accessory pathway. Isoproterenol was not employed as all children were tachycardic after insertion of the transesophageal catheter. If the atrial extrastimuli were unsuccessful for atrial fibrillation induction, burst pacing at 150 ms was used for up to 15 s with the intention to induce atrial fibrillation with a duration of more than 10 s.

Conduction over the accessory pathway was also evaluated during atrial pacing by finding the shortest atrial cycle length for which there was 1-to-1 conduction over the accessory pathway.

In children younger than 12 years of age, electrophysiological evaluation was performed under sedation with Propofol, following established pediatric protocols. Sedation was used to minimize discomfort and facilitate safe transesophageal catheter placement. Children aged 12 years and older underwent the procedure without sedation. The potential influence of sedation on atrial vulnerability and the inducibility of atrial fibrillation was considered, as Propofol can alter autonomic tone and electrophysiological properties.

### 2.3. Definitions

In case of an effective refractory period of the accessory pathway less than 250 ms or shortest pre-excited RR interval during atrial fibrillation of less than 250 ms or AVRT induction, or minimal cycle length with 1:1 conduction through the accessory pathway < 250 ms, the accessory pathway was considered to be at high risk and had an invasive EP study with radiofrequency ablation of the accessory pathways. Patients in whom arrhythmias were not induced and had an APERP > 250 ms or minimal cycle length with 1:1 conduction through the accessory pathway > 250 ms were considered to be at low risk.

### 2.4. Statistics

For continuous data, we used means and standard deviations, and for comparison, the independent sample Student T test. For categorical data, we used percentages, and for comparison, the chi-square test. Bland–Altman plots of differences were used to compare 2 ways of expressing high risk, and we plotted on a scatter plot the mean and 95% confidence intervals. For all analyses, we used the SPSS package for Windows, version 23 SPSS Inc. (Chicago, IL, USA), IBM, and *p* < 0.05 was considered to indicate a significant difference.

## 3. Results

Between April 2022 and December 2025, we carried out 41 transesophageal electrophysiological studies in 41 patients with asymptomatic WPW ranging in age from 1 to 18 years old (mean age 12.5 +/− 4.4 years). Children older than 6 years underwent a treadmill stress test to determine the maximum heart rate and evaluate atrioventricular conduction. Only children without an abrupt or complete loss of pre-excitation during the test were included in this study. For children younger than 6 years, Holter ECG was the sole non-invasive assessment performed prior to transesophageal electrophysiological evaluation.

Of the 41 children, 15 (36.6%) were less than 12 years old, and 26 (63.4%) were more than 12 years old. Transesophageal electrophysiological studies were performed under Propofol sedation in all children younger than 12 years (*n* = 15) and without sedation in children aged 12 years and older. Among younger children, atrial vulnerability and inducibility of atrial fibrillation were observed less frequently (1 child out of 15) compared with older children, consistent with differences in age-related atrial properties. The localization of the accessory pathway assessed in maximal pre-excitation was left lateral in 10 cases, right lateral in 2 cases, posteroseptal (left or right) in 20 cases, and anteroseptal in 9 cases.

An orthodromic reentrant tachycardia was induced in four children, two of them being without any symptoms during tachycardia (Table 1). Twenty-nine children had a benign form of pre-excitation, the APERP, and the shortest RR interval during pre-excited AF was superior to 250 ms. In children practicing sports, we authorize sports activities without any limitations. The remaining twelve patients had high-risk accessory pathways, and in those children, an invasive intracardiac electrophysiological study with catheter ablation was performed.

**Figure 1:**
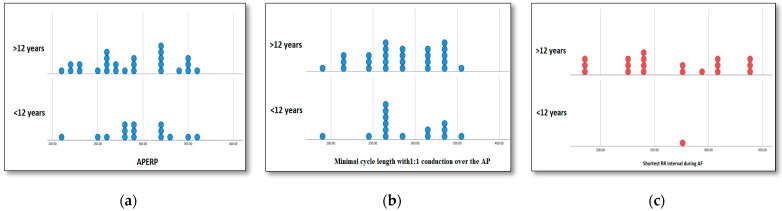
Values obtained during electrophysiological studies. (**a**) Accessory pathway effective refractory period in children above and below 12 years of age. (**b**) Minimal cycle length 1-to-1 conduction over the accessory pathway in children above and below 12 years of age. (**c**) Shortest RR interval between 2 consecutive pre-excited beats during atrial fibrillation. APERP = accessory pathway effective refractory period; AP = accessory pathway; and AF = atrial fibrillation.

The mean APERP in the under-12-year-old group was 296 +/− 38 ms, while in the group over 12 years of age, it was 286 +/− 45 ms without a significant difference between groups (*p* = 0.48). The mean 1:1 conduction over the accessory pathway was 287.3 +/− 41.5 ms in the < 12-year-old group compared to 282.4 +/− 45.7 ms in the >12-year-old group (*p* = 0.71). Fourteen patients had inducible atrial fibrillation: in the <12-year-old group, only 1 child of 11 years had inducible atrial fibrillation, while in the group of children > 12 years old, 13 (50%) had inducible atrial fibrillation (*p* < 0.0001) (Figure 1). In the remaining patients, atrial fibrillation could not be induced by burst pacing up to 150 ms. Based on the three above-mentioned values, eleven patients had high-risk accessory pathways in the >12-year-old group and one in the <12-year-old group. (*p* = 0.016).

**Figure 2:**
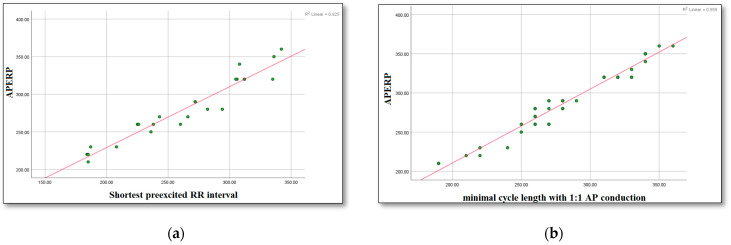
(**a**) Correlation between APERP and the shortest pre-excited RR interval during atrial fibrillation, where R^2^ = 0.925. (**b**) Correlation between APERP and minimal cycle length showing 1:1 conduction over the accessory pathway, where R^2^ = 0.959.

**Figure 3:**
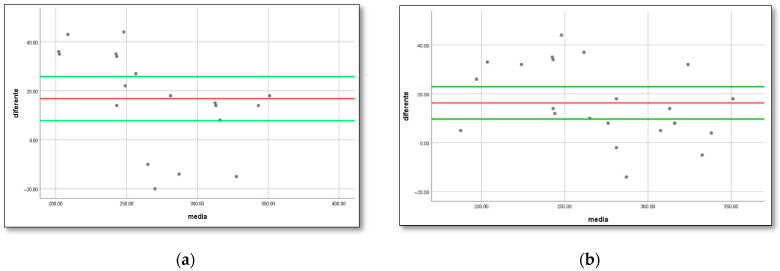
(**a**) Shortest pre-excited RR interval during atrial fibrillation vs. APERP. The upper and lower limits of the 95% confidence intervals (7.8–25.8) are marked with a green line, and the mean difference (16.8) is marked with a red line. There is a significant proportional bias with points outside the 95% confidence interval lines. (**b**) The shortest pre-excited RR interval during atrial fibrillation vs. minimal cycle length showing 1-to-1 conduction over the accessory pathway. The upper and lower limits of the 95% confidence intervals (9.7–22.9) are marked with a green line, and the mean difference (16.3) is marked with a red line. There is a significant proportional bias with points outside the 95% confidence interval lines.

A significant (*p* < 0.001) correlation (r = 0.91) was found between the APERP and the shortest RR interval between two consecutive pre-excited beats during AF. A significant (*p* < 0.001) correlation (r = 0.96) was also found between the APERP and the shortest 1:1 conduction over the accessory pathway (Figure 2). Nonetheless, substantial variations (Figure 2) were observed in certain cases: of the 12 patients with a short RR interval between consecutive pre-excited beats during an AF of 250 ms, the anterograde effective refractory period of the accessory pathway was found to be >260 ms in three patients (25%). Conversely, of the 17 patients with a relatively long APERP (>250 ms), 3 (17.6%) had a short RR interval between consecutive pre-excited beats during AF (<250 ms). The Bland Altman plots of differences and means between the 2 measurement techniques assessing the risk of the accessory pathway: APERP vs. shortest pre-excited RR interval during atrial fibrillation and minimal cycle length showing 1:1 conduction over the accessory pathway vs. shortest pre-excited RR, showed significant proportional bias with points outside the 95% confidence interval lines (Figure 3).

During follow-up, no patient experienced syncope or sudden cardiac death. Eleven children subsequently underwent intracardiac electrophysiological study with catheter ablation of the accessory pathway, with an immediate procedural success rate of 100%. Two patients experienced recurrence at three- and ten-month follow-up, respectively, and required repeat ablation.

## 4. Discussion

Our findings indicate that orthodromic reentrant tachycardia (ORT) and atrial fibrillation occur less frequently in children under 12 years of age, attributed to both the use of sedation and the inherent characteristics of the smaller atrium, which is less susceptible to arrhythmia compared to children over 12 years of age. This cutoff was selected based on prior electrophysiological studies, including those by Brembilla-Perot et al. [9,10], in which 12 years is commonly used as the age distinguishing sedation versus non-sedation during electrophysiological studies. In addition to this practical consideration, younger age is associated with physiological factors relevant to atrial fibrillation inducibility, including smaller atrial size, longer atrial effective refractory periods, and a lower likelihood of sustaining atrial fibrillation. These observations are consistent with the “critical mass” theory [11,12,13,14], which proposes that a minimum atrial mass is required to maintain fibrillatory activity and has been demonstrated in experimental and computational models. While autonomic tone differs in children compared with adults [15], atrial size appears to play a more prominent role in atrial fibrillation inducibility. In addition, developmental changes in accessory pathways further support the age-based stratification. Morphopathological studies have demonstrated that accessory atrioventricular connections are more prevalent during fetal life and early infancy and decrease in number with maturation of the atrioventricular fibrous rings [16,17]. When accessory pathways persist beyond infancy, those with longer effective refractory periods appear more likely to lose anterograde conduction with increasing age.

For children under 12 years, assessing risk with a transesophageal EP study is preferable to an invasive intracardiac study. Most of them necessitate sedation; nevertheless, a research by Brembilla-Perot et al. indicated that sedation may be avoided in children aged 5 to 12 years old [9,10]. However, for the comfort of physicians and children, we opted for sedation, which extends the transesophageal EP procedure by 10 to 30 min, considering peripheral venous line placement, sedative drug administration, and the additional time required for patient recovery.

All 41 children from our study were asymptomatic. However, nearly 30% of asymptomatic patients may exhibit symptoms attributable to arrhythmia throughout a 12-year follow-up period [18]. Some patients may exhibit ventricular fibrillation as the initial manifestation. Motoya and Timmermans found that 26% and 53% of their patients, respectively, suffered ventricular fibrillation as the initial symptom of WPW syndrome [1,19]. Stress tests and Holter ECG demonstrate insufficient specificity for identifying patients at elevated risk of sudden death [20,21]. Electrophysiological studies have proven to be an efficient tool for evaluating the risk of sudden death, with a transesophageal approach in asymptomatic children and the invasive intracardiac method in symptomatic children and adults [9]. Wellens et al. compared the APERP with the ventricular rate during spontaneous or induced atrial fibrillation and identified a strong association between the two variables [22].

The transesophageal approach is the preferred technique for children under 12 years of age. The intracardiac approach presents several complications in pediatric patients, which may be unacceptable for an asymptomatic child. Some parents may decline the intracardiac invasive procedure. In the study conducted by Pappone et al. on asymptomatic children, 10 out of 60 patients in the high-risk group declined catheter ablation [23]. In their cohort, there were 5 cases of pneumothorax following a superior approach via the subclavian vein, femoral hematoma in 25 children, arteriovenous fistula in 2, right bundle branch block in 10, left bundle branch block in 3, minor pericardial effusion in 2 children, and third-degree atrioventricular block in 1 child. Additional problems include radiation exposure lasting from minutes to hours, a required 48 h withdrawal from school, and a restriction to vigorous exercise 7 to 14 days following venous or arterial femoral puncture to prevent hemorrhage or vascular complications. Electrophysiological study and anesthesia present a significant risk, affecting 6% of children, while the ablation technique provides a supplementary risk of 15%. The risk of complications increased in children below the age of 10 years [24]. Is the transesophageal study a viable alternative to the invasive intracardiac procedure in pediatric patients under 12 years old? It certainly is, providing similar results. Samson et al. demonstrated that the findings of transesophageal electrophysiological studies and intracardiac electrophysiological studies are comparable [25].

We induced orthodromic reentrant tachycardia (ORT) in four children: all from the over-12-years cohort and none from the under-12-years cohort. This may be attributable to our methodology of stimulation, utilizing only one extrastimulus and avoiding isoprenaline administration. We employed the identical methodology as Milstein et al., who administered one extrastimulus and excluded isoprenaline from their protocol [26]. Nonetheless, the PACS/HRS Consensus indicates that the risk profile in asymptomatic WPW is determined at baseline [27]. Hence, Dubin et al. also refrained from using isoprenaline in their investigation. The second issue that may have affected our results is sedation in children under 12 years, which could have reduced stress hormones and rendered arrhythmia uninducible [28]. Our findings align with those of Brembilla et al., who reported that only 3 out of 40 patients (8%) were inducible with programmed atrial stimulation in their cohort of children [29]. Sarrubi et al. reported a higher incidence of inducible ORT of 53% in a cohort of 62 children [30]. Moreover, in the research conducted by Dubin et al., over 50 percent of their asymptomatic patients exhibited inducible ORT during the electrophysiologic examination, and 2 out of 23 individuals presented antidromic reentrant tachycardia [28]. Inducible ORT may be a risk factor for ventricular fibrillation. Atrial vulnerability increases at high pacing rates, and it is probable that the accelerated rates observed during orthodromic tachycardia contribute to the progression of tachycardia to atrial fibrillation [31]. In the research conducted by Klein et al., a history of reciprocating tachycardia was more common in the cohort showing ventricular fibrillation. ORT deteriorated in atrial fibrillation in 4 out of 25 individuals from the VFib group [7]. This may also explain the findings from the study of Pappone et al., which reported a significant prevalence of asymptomatic children with inducible tachycardia (36.4%) complicated with one sudden death and two ventricular fibrillations in children refusing catheter ablation [23].

We induced atrial fibrillation in 34% of our patients, 50% from the >12 years old group, and only 1 of the children from the group below 12 years of age. Eight children from the >12-year-old group had the shortest pre-excited RR interval during AF < 250 ms, and none from the <12-year-old group. The inability to induce atrial fibrillation in many of our children under 12 years old may be attributed to the lack of atrial electrical instability due to their young age, the reduced size of the left atrium, and the use of sedation, which reduces plasma catecholamine levels [32]. Prior research indicated that the risk of ventricular fibrillation and sudden cardiac death was significantly elevated in patients with WPW syndrome exhibiting the shortest pre-excited RR interval of less than 250 ms during atrial fibrillation [7]. The mean ventricular rate during induced atrial fibrillation is slightly slower than that observed during spontaneous arrhythmia; yet, a strong linear correlation between the two measurements has confirmed the efficacy of measurements made during the electrophysiological study [33]. Our findings align with those of Hoyt et al., who adopted a stimulation protocol involving decremental atrial pacing at a cycle length of 140 ms for a duration of up to 30 s. AF inducibility was defined by a duration exceeding 20 s. This procedure yielded an inducibility rate of 88.5% (23 out of 26) in children with a mean age of 11.9 years [34]. We used a similar stimulation protocol and obtained an inducibility rate of 1 child out of 15 in the under-12-year-old group and 13 out of 26 children from the over-12-year-old group. We defined all atrial events lasting longer than 10 s as inducible atrial fibrillation. Atrial fibrillation is rarely induced in children under 10 years of age. In the study of Brembilla-Perot et al. on 51 children aged 6 to 19 years old, atrial fibrillation was induced in 12 children (22%): 0 in the group of 6 to 10 years old, 8 in the group of 11 to 15 years old, and 4 in the group of 16 to 19 years old [9]. However, in the study of Hoyt et al. authors could also induce atrial fibrillation in four children younger than 6 years old (age 2 to 6 years). A 5-year-old child exhibited a high-risk accessory pathway, with the shortest RR interval of 220 ms, whereas the other three children had the shortest RR intervals exceeding 250 ms.

Although there was a significant correlation between the shortest RR interval during AF and AERP and the shortest cycle length showing 1:1 conduction over the AP (SCL1:1AP), Bland–Altman graphs showed significant proportional bias, with several points outside the 95% confidence interval lines on the graph of differences. This is in line with the study of Klein et al., showing that the shortest cycle length with 1-to-1 conduction over the accessory pathway was not similar to APERP, although there was a strong correlation between the values. In their cohort, one patient from the VF group had an SCL1:1AP value below 200 ms, while four patients had APERP below 200 ms. Furthermore, none of the patients had SCL1:1 AP over 300 ms, while two patients had APERP over 300 ms [7].

Our patients ranged in age from 1 to 18 years. We concur with Dr. Brembilla-Perot et al. that the transesophageal examination should be conducted immediately following detection of the WPW pattern on the ECG of an asymptomatic child [10]. The PACES/HRS Expert consensus on the management of the asymptomatic young patient with a Wolff–Parkinson–White electrocardiographic pattern recommends utilization of transesophageal or intracardiac EP study to assess the risk of the accessory pathway in individuals 8 to 21 years whose noninvasive testing did not demonstrate a clear and abrupt loss of pre-excitation (Class 2A, LOE B/C). SPERRI < 250 ms in atrial fibrillation are at an increased risk for sudden cardiac death, and catheter ablation is reasonable (Class 2A, LOE B/C.). SPERRI > 250 ms in atrial fibrillation are at a lower risk of sudden cardiac death, and it is reasonable to defer catheter ablation (Class 2A, LOE C.).

### Limitations

Several technical limitations must be acknowledged in our low-number cohort of children: (1) The precise measurement of the APERP is occasionally unachievable because the left atrium becomes refractory prior to the block in the accessory pathway. (2) The APERP changes with the cycle length. A fixed rate of 500 ms was used; however, the most valid APERP measurements would potentially be established at cycle lengths similar to those seen in orthodromic reentrant tachycardias. (3). We collected our APERP and minimal cycle duration with 1:1 conduction on a 10 ms scale, since we applied extrastimuli at 10 ms decrement. However, while determining the shortest RR interval during AF, more accurate measurements were obtained utilizing calipers with a 1 ms scale. (4). The use of Propofol sedation in children under 12 years represents a potential confounding factor in the interpretation of the inducibility of atrial fibrillation. Sedation can alter autonomic tone and atrial electrophysiological properties, which may have contributed to the lower incidence of inducible atrial fibrillation observed in younger children. While our findings suggest age-related differences in atrial vulnerability, these results should be interpreted with caution, as the effects of sedation cannot be fully separated from the developmental differences in atrial size and electrophysiological properties. This limitation highlights the need for cautious extrapolation of these results and supports the hypothesis-generating nature of the study rather than definitive guideline-level recommendations. A better option would be Midazolam, which modulates autonomic tone and atrioventricular conduction; however, its effects on atrial vulnerability and inducibility of atrial fibrillation are modest compared to Propofol. (5) Due to the limited sample size of our study, 41 children (15 vs. 26 per group), it may offer sufficient power only for moderate to large effects but not enough power to identify minor differences between the two groups. Potentially clinically significant differences cannot be ruled out even if we found no differences between the two groups in terms of APERP, 1:1 conduction over the accessory pathway, or the shortest pre-excited RR interval during atrial fibrillation. Therefore, the lack of statistically significant differences should be viewed cautiously, and our results should be regarded as hypothesis-generating until larger, sufficiently powered studies validate them.

## 5. Conclusions

Asymptomatic children with a WPW pattern under 12 years of age, evaluated by transesophageal electrophysiological study, showed a reduced incidence of inducible atrial fibrillation compared to those older than 12 years. Moreover, they had accessory pathways with a lower risk, and none of the children under 12 years required catheter ablation. Despite the strong correlation among the three high-risk values, discrepancies were noted in the measurements of APERP, with a minimum cycle length of 1:1 conduction through the accessory pathway, and the shortest RR interval during pre-excited atrial fibrillation; therefore, all factors must be considered when recommending catheter ablation of the accessory pathway. While the results support the use of transesophageal electrophysiological study in younger, asymptomatic children following identification of a Wolff–Parkinson–White pattern on the ECG, they should be interpreted as hypothesis-generating and are not sufficient to justify changes to current guideline recommendations.

## Figures and Tables

**Table 1:** Clinical and electrophysiological findings in the children’s cohort.

	Age < 12	Age > 12	
Asymptomatic	15	26	
Symptomatic	0	0	
Age (years)	7.5 +/− 2.5	15.5 +/− 1.9	**<0.0001**
Female sex (%)	50%	76%	*p* = 0.08
Mean shortest cycle length of 1:1 AP (ms)	287 +/− 41	282 +/− 46	*p* = 0.71
Mean APERP (ms)	296 +/− 38	286 +/− 45	*p* = 0.48
Mean shortest RR during AF (ms)	280	262 +/− 51	*p* = 0.75
Inducible PSVT	0	4	*p* = 0.11
Inducible AF	1	13	***p*** **< 0.001**
High risk necessitating catheter ablation	1	11	***p*** **= 0.016**

## Data Availability

The data supporting reported results can be found archived in Mega Cloud and are available at the following link: https://mega.nz/fm/IZoUGCaQ (accessed on 3 December 2025).

## Abbreviations

The following abbreviations are used in this manuscript:

AP	Accessory pathway
APERP	Accessory pathway effective refractory period
AF	Atrial fibrillation
WPW	Wolff–Parkinson–White
